# A Clinical Retrospective Study on the Transmission of COVID-19 From Mothers to Their Newborn and Its Outcome

**DOI:** 10.7759/cureus.20963

**Published:** 2022-01-05

**Authors:** Rajangam Ponprabha, Srinivasan Thiagarajan, Kandan Balamurugesan, Prem Davis

**Affiliations:** 1 Pediatrics, Government Villupuram Medical College, Villupuram, IND; 2 Pediatrics, Indira Gandhi Medical College and Research Institute, Puducherry, IND; 3 Medicine, Jawaharlal Institute of Postgraduate Medical Education and Research, Puducherry, IND; 4 Health Centre, Central University of Tamil Nadu, Thiruvarur, IND; 5 Otorhinolaryngology, Head and Neck Surgery, Sri Venkateshwaraa Medical College Hospital and Research Centre, Puducherry, IND

**Keywords:** newborn, breastfeeding, vaginal delivery, vertical transmission, covid-19

## Abstract

Introduction

India is the second most populated country in the world. The declaration of the COVID-19 pandemic has caused significant morbidity and mortality in pregnant women and newborns. Due to the decreased lung volume and immunocompromised state, pregnant women are more prone to rapid clinical deterioration. Regarding the transmission of COVID-19 infection to newborns, there is no clear-cut evidence regarding the intrauterine or vertical transmission of COVID-19 from the affected pregnant women to their neonates.

Aim

This study aimed to assess the outcome of neonates born to mothers with COVID-19 in a tertiary care hospital in Puducherry, India.

Methods

This retrospective case record-based study was conducted among all neonates born to COVID-19-positive mothers at a tertiary care institution in Puducherry from April 2020 to June 2020. All the newborns born to COVID-19-positive mothers during the specified period of time were included in the study.

Results

A total of 98 COVID-positive mothers were included in the study. Of these, 13.27% showed mild upper respiratory infection, and one had a moderate category. The mean gestational age was 38.4+1.12 weeks. About 53.06% of COVID-19-positive mothers had C-sections, 45.92% had a normal vaginal delivery, and only one had an instrumental vaginal delivery. All the mothers gave birth to singleton neonates; of 98 newborns, 51 were female, and 47 were male with the mean birth weight of 3.1 ± 0.4 kg. Among the 98 neonates, only six developed COVID-19 infection. Among the six, four acquired infections during the first to second week of the postnatal period and two acquired infections during the fourth week. Three neonates born by normal vaginal delivery only had a fever; two neonates manifested fever, cough, and increased respiratory rate; and only one neonate developed hypoxia.

Conclusion

The study showed that vertical transmission from the mother to the newborn is extremely minimal. In our study, six neonates acquired infection mostly due to the close contact of the newborn with the mother during rooming-in and breastfeeding.

## Introduction

India is the second most populated country in the world with a fertility rate of 2.179 births per woman [1,2]. With the birth of 25 million children yearly, India contributes to one-fifth of the world’s annual birth rate, which is largest in the world [3]. The coronavirus disease 2019 (COVID-19) outbreak is the third outbreak of the coronavirus group after severe acute respiratory syndrome coronavirus (SARS-CoV) outbreaks in 2002 and Middle East respiratory syndrome coronavirus (MERS-CoV) outbreaks in 2012 [4-6]. Both infections had caused a significant morbidity and mortality to pregnant women and their newborns [7,8]. In the wake of the COVID-19 outbreak in India, pregnant women are facing a new challenge in their lives. Moreover, they are at high risk of health and mental problems caused directly or indirectly by the COVID-19 infection, considering that COVID-19 seems to have a similar pathogenic potential as SARS-CoV and MERS-CoV.

From the declaration of the COVID-19 pandemic by the World Health Organization (WHO) on March 11, 2020, India is one of the most affected countries globally, ranking second in the world, with a case number of 27,547,705, and first among the Asian countries [9,10]. Moreover, COVID-19 infection and death rates among pregnant women are also increasing compared to those in nonpregnant women. Recent data from the Centers for Disease Control and Prevention (CDC) report that pregnant women are more likely to have severe disease and therefore have higher risk of ICU admissions and mechanical ventilation because they are in a particular state of immune suppression and more susceptible to respiratory pathogens. Because of decreased lung volume caused by increases in uterus size during pregnancy, patients might be more prone to more rapid clinical deterioration with COVID-19 during pregnancy, which may increase the risk of adverse pregnancy outcomes [11,12].

The risk of maternal mortality is 22 times higher than that in pregnant women without COVID-19 [13]. Presently, there are no data to imply the direct severe impact on their newborn. Regarding the transmission of the COVID-19 infection, there are many conflicting studies on intrauterine or vertical transmission of COVID-19 from affected pregnant women to their neonates although the overall rate is reportedly extremely low [14,15]. Moreover, there is no clear-cut evidence on virus transmission via breastmilk. Most studies have not demonstrated the virus in breastmilk, so breastfeeding recommendations have not changed, but viral particles have been demonstrated in breastmilk [16,17]. From the reports of the CDC, it is evident that there is no difference in the risk of infection between neonates who were roomed-in with the mother and those cared for in separate rooms [18]. Despite these factors, which show that the spread of infection from the mother to the child is low, there is an increased risk of transmission of infection to the neonate from the mother, unless adequate infection prevention measures are followed by the mother. Given these set of factors, neonates born from mothers with COVID-19 have to face unique challenges in this present pandemic. Currently, there are no studies and guidelines available in India and worldwide for the abovementioned problem. Thus, this study aimed to assess the outcome of neonates born to mothers with COVID-19 in India.

## Materials and methods

Study design

This retrospective case record-based study was conducted among all neonates born to COVID-19-positive mothers at a tertiary care institution in Puducherry from April 2020 to June 2020.

Sampling

During the abovementioned period, all pregnant women in labor or who are likely to deliver in the next five days, residing in clusters/containment areas or in large migration gathering centers or from hotspot district, with positive test for severe acute respiratory syndrome coronavirus-2 (SARS-CoV-2) infection tested by real-time reverse transcriptase-polymerase chain reaction (RT-PCR) of nasopharyngeal or throat swab were included in the study.

Data collection

After obtaining appropriate informed consent, the mothers who tested positive for SARS-CoV-2 were included in the study. Basic demographic details of the mother with antenatal history, blood group, comorbidities, pregnancy-related complications, delivery mode, and any SARS-CoV-2 symptoms of the mother, if present, were collected. After the delivery, neonates of the study participants were tested for COVID-19 by RT-PCR of nasopharyngeal swab after 24 hours of birth, and the next swab was obtained between the 5th and 10th day, depending upon the development of the symptom and signs of the newborn. Based on the development of the symptoms, the neonates were classified into mild, moderate, and severe categories based on the guidelines issued by Ministry of Health and Family Welfare, Government of India. Based on the guidelines, the patients with fever, sore throat, malaise, and rhinorrhea were classified as mild category; the patients with fast breathing (i.e., more than 60 breaths per minute for children less than two months of age) were classified under moderate category; and the patients with symptoms of sepsis, acute respiratory distress syndrome, shock, cyanosis, decreased peripheral capillary oxygen saturation (SPO_2_) below 94%, increased respiratory effort, lethargy, somnolence, and seizure were categorized into severe category [19]. All mothers were advised to perform direct breastfeeding irrespective of their COVID-19 status, with proper hand hygiene and cough etiquette. Data on neonatal birth weight, feeding pattern, Appearance, Pulse, Grimace, Activity, and Respiration (APGAR) score, investigations, and neonatal outcomes were collected. All newborns with their mothers were on constant monitoring during the hospital stay. Moreover, all newborns with the mother were discharged on the 10th postnatal day. Telephone follow-up of COVID-19-positive neonates was conducted until one month of age. 

Data analysis

All data were entered in Microsoft Excel (Microsoft Corporation, Redmond, Washington, USA) and analyzed using Epi Info 7.0 version (Centers for Disease Control and Prevention (CDC), Atlanta, Georgia, USA) for Windows. Descriptive statistics including frequencies, percentages, mean, and standard deviations were calculated. Inferential statistics were applied based on the nature and distribution of the data.

Ethical approval

The study protocol was approved by the Internal Human Ethics Committee of the Indira Gandhi Medical College and Research Institute, Puducherry, India, with reference number Pro No.259/IEC No-29/PP/2020.

## Results

Since all mothers gave birth to a singleton newborn, a total of 98 newborns were included in the study, of which six (6.12%) tested positive for RT-PCR and 92 (93.88%) tested negative.

Clinical profile of the mothers

Of the 98 COVID-19-positive mothers, 84 (85.71%) did not manifest any symptoms of COVID-19 infection, 13 (13.27%) manifest mild upper respiratory tract infection symptoms and had mild COVID-19 infection, and only one (1.02%) had moderate category with symptoms of fever, sore throat, and mild hypoxia. The mean gestational age of the mothers was 38.4 ± 1.12 weeks. A majority of the mothers (52 [53.06%]) had the blood type “O” positive, and 23 (24.21%) mothers had blood types of “A” and “B” positive. Regarding the comorbidities of the mother, eight (8.17%) mothers were diagnosed with gestational diabetes mellitus, five (5.1%) were diagnosed with pregnancy-induced hypertension, and only four (4.08%) had hypothyroidism. Moreover, 14 (14.29%) mothers had history of lower (uterine) segment cesarean section (LSCS) of various causes.

In the present pregnancy, 52 (53.06%) COVID-19-positive mothers had C-section, 45 (45.92%) had normal vaginal delivery, and only one (1.02%) had instrumental vaginal delivery. Regarding the various reasons for LSCS among COVID-19-positive mothers, 14 (14.29%) had history of LSCS, seven (7.14%) had premature rupture of membrane, six (6.12%) had fetal distress, five (5.1%) had oligohydramnios, three (3.06%) had their fetus in breech presentation, two (2.04%) had unstable lie of the neonate, and only two (2.04%) had failed induction during delivery. Regarding the feeding pattern of the mother, a majority of the mothers (59 [60.2%]) fed their neonates with expressed breastmilk, 38 (38.78%) mothers performed direct breastfeeding, and only one (1.02%) neonate required intravenous (IV) fluid initially. The IV fluid was initiated because the neonate developed respiratory distress and aspiration; then it was shifted to direct breastfeeding on the fifth postnatal day. Of the six COVID-19-positive neonates, three received direct breastfeeding.

Clinical profile of the neonates

Of 98 newborns, 51 (52.04%) were female and 47 (47.96%) were male. Most neonates (92 [93.88%]) were born at term, only three (3.06%) were born at preterm, and three (3.06%) were born at postterm. The mean birth weight was 3.1 ± 0.4 kg, and all neonates had an APGAR score of > 7/10 at one and five minutes time period. The average duration of COVID-19 positivity from mother to newborn was 11 days, and the mean postnatal age of COVID-19 positivity in neonates was 4.8 days. The mean respiratory rate among the neonates was 52, and the mean SPO_2_ was 98% at room air. Twelve (12.2%) neonates received IV antibiotics for sepsis screening positive neonates and those with respiratory distress or tachypnea. Moreover, 40 (40%) neonates had neutrophilia, 12 (12.2%) had lymphocytosis, three (3.06%) had eosinophilia, and 10 (10.2%) had thrombocytopenia. Leukocytosis was noted in 70 (71.4%) neonates, among them five were positive for COVID-19. The case series of the six neonates with COVID-19 is presented in Table 1.

**Table 1:** Characteristics of neonatal clinical profile CPAP: continuous positive airway pressure, WNL: within normal limits, GGO: ground-glass opacity, SPO2: peripheral capillary oxygen saturation.

Variable	Patient 1	Patient 2	Patient 3	Patient 4	Patient 5	Patient 6
Age of the newborn (days) at admission	25	27	17	12	6	13
Type of delivery	Vaginal delivery	Vaginal delivery	Vaginal delivery	C-section	C-section	C-section
Birth weight (kg)	2.61	3.1	3.62	2.56	2.55	3.01
Type of feeding	Expressed breast milk	Expressed breast milk	Direct breastfeeding	Direct breastfeeding	Direct breastfeeding	Expressed breast milk
Respiratory rate	56	49	44	52	62	70
SPO_2_	98	96	98	98	98	98
Chest radiography	WNL	WNL	WNL	WNL	Bilateral basal haziness (+)	WNL
CT	No significant abnormalities suggestive of COVID-19 pneumonia	No significant abnormalities suggestive of COVID-19 pneumonia	No significant abnormalities suggestive of COVID-19 pneumonia	No significant abnormalities suggestive of COVID-19 pneumonia	Subpleural patchy GGO in the left lower basal segments consistent with early COVID-19 pneumonia	No significant abnormalities suggestive of COVID-19 pneumonia
Leukocyte count (8,000 to 15,000 cells/µL)	24,600	34,700	16,300	24,700	12,300	8,700
Lymphocyte count (3,000–8,000 cells/µL)	21%	18%	51%	27%	27%	46%
Platelet count (1.5 lakh–5 lakh cells/µL)	197,000	193,000	118,000	234,000	225,000	342,000
Symptoms	Fever	Fever	Fever	Fever, cough, increased respiratory rate	Fever, cough, increased respiratory rate, hypoxia	Fever, cough, increased respiratory rate
Ventilation	Nil	Hood	Nil	Hood	CPAP	Hood
Antibiotic therapy	Yes	Yes	Yes	Yes	Yes	Yes

**Figure 1:**
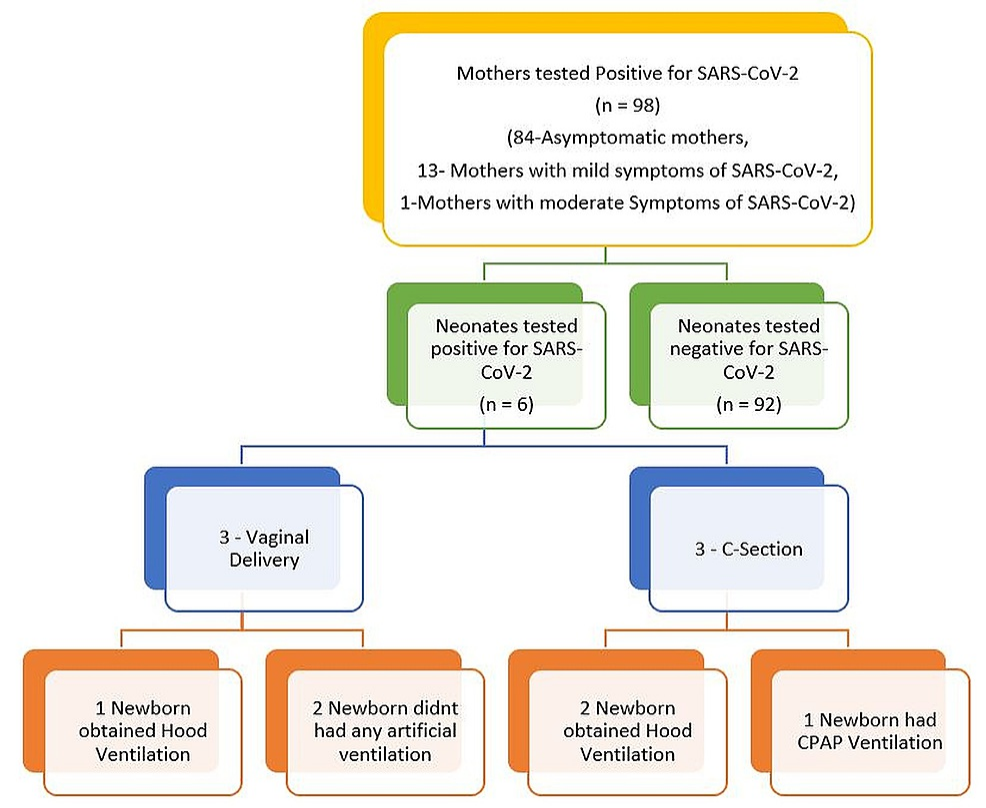
Study profile regarding the transmission of COVID-19 infection from mother to neonates SARS-CoV-2: severe acute respiratory syndrome coronavirus-2; CPAP: continuous positive airway pressure.

Of the six COVID-19-positive neonates, four acquired infection during the first to second week of the postnatal period and two acquired infection during the fourth week. Moreover, of six COVID-19-positive neonates, three were born by vaginal delivery and three by C-section. Two of the three neonates born by C-section received direct breastfeeding, and only one neonate born by vaginal delivery received direct breastfeeding. The neonates born by normal vaginal delivery had symptoms of fever, but among the neonates born by C-section, two developed fever, cough, and increased respiratory rate and one developed hypoxia in addition to fever, cough, and increased respiratory rate. All three neonates born by C-section received some artificial ventilation, via oxygen hood for two neonates and continuous positive airway pressure (CPAP) for one neonate (Figure 1). Only one neonate born by vaginal delivery received oxygen hood ventilation. Chest radiography and computed tomography (CT) were performed in all COVID-19-positive neonates, in which one neonate who was born by C-section had bilateral basal haziness in chest radiography and subpleural patchy ground-glass opacity in left lower basal segments in CT, which was suggestive of COVID-19 development in a neonate. 

## Discussion

In this study, all COVID-19-positive mothers gave birth to a single-term fetus, and there are no stillbirths in our study. In our study, of 98 newborns, six were positive for COVID-19. Thus, the overall infection rate was 6.12% for the neonates born to COVID-19-positive mothers. The study from Yale shows that the vertical transmission rate is 3.2%, which is much lower than that in our study, but our study was conducted in a smaller population compared to that in the Yale study [20]. Although pregnancy is an immunosuppressive state and pregnant women are highly susceptible to respiratory pathogens and pneumonia, in our study, a majority (85.71%) of the pregnant women did not have any COVID-19 symptoms and 1.02% of pregnant women showed moderate COVID-19 symptoms. However, the study conducted in New Brunswick, New Jersey, shows that 88.5% of the pregnant women manifest mild disease and only 1.6% of pregnant women manifest severe COVID-19 infection [21]. The abovementioned finding of mild COVID-19 infection among pregnant women is comparable to existing data on severity of COVID-19 infection in pregnant women. In our study, most neonates received breastmilk either directly or through expressed milk, except for one infant. Since no study has found virus transmission through breastmilk, all neonates received breastfeeding from the day of birth [22]. In our study, a majority of the pregnant women had normal vaginal delivery and only 14.29% underwent C-section due to previous and present obstetrical causes. The study from China also shows that there is no difference in neonatal COVID-19 infection, neonatal deaths, and maternal deaths between vaginal delivery and C-section. Moreover, the same study also insisted that the mode of the birth of the newborn should be individualized only based on the disease severity and obstetric indications [23].

In our study, all neonates had an APGAR score of not less than 7 at one and five minutes, which is similar to the study conducted in Mumbai, India, which shows that 97.7% of the neonates born to the COVID-19-positive mothers have an APGAR score of 7-10. From this, we found that COVID-19 infection did not affect the general condition of the newborn at birth [24]. Similar to the study in Mumbai, India, where the birth weight of the newborns of COVID-19-positive and COVID-19-negative mothers ranges from 2.5 to 2.9 kg, our study also shows the mean birth weight of 3.1 ± 0.4 kg [24]. In our study, 11 days is the average duration of the mother-to-neonate positivity and the mean neonatal COVID-19 positivity was 4.8 days. Moreover, among six COVID-19-positive newborns, four acquired infection during the first to second week and two acquired infection during the fourth week of the postnatal period. Thus, none of the neonates were positive in the first swab test, which was obtained within 24 hours of birth. There are insufficient data to compare the abovementioned parameter. Therefore, it is very clear that none of the newborns were positive at the time of birth, suggesting that the vertical transmission of the COVID-19 from the mother to newborn is low [25]. Among the six COVID-19-positive newborns, four (66.67%) received artificial ventilation either through oxygen hood or via CPAP because of the development of the respiratory distress. Moreover, the proportion of acquiring artificial ventilation is higher among the neonates born by C-section than those born through normal vaginal delivery. Because the three neonates who were born by C-section received artificial ventilation compared to neonates born by normal vaginal delivery, only one neonate received artificial ventilation via oxygen hood. The Korean study also shows that 22.4% of the newborns developed respiratory distress and received mechanical ventilation [26]. In our study, two of the six COVID-19-positive newborns developed respiratory distress and were admitted to the neonatal intensive care unit (NICU). This is in contrast to the finding of the Mumbai study, where only 20.14% of the newborns developed intrauterine fetal distress and admitted to the NICU for management [7]. In our study, no mortality had been reported among the COVID-19-positive neonates. The New York study also shows a similar result regarding the neonatal mortality, with no invasive mechanical ventilation and neonatal sepsis [27].

## Conclusions

The vertical transmission rate of COVID-19 infection from the mother to neonate is extremely minimal. In our study, six neonates acquired infection mostly due to the close contact of the newborn with the mother during rooming-in and breastfeeding. There is no sufficient evidence to support that C-section is better than vaginal delivery in preventing the vertical transmission of COVID-19 from the mother to neonate, and the mode of birth should be based on the individual obstetric indications only. We recommend that there should be proper spacing between the newborn and mother and breastfeeding should be provided with proper COVID-19 precautions, such as wearing the mask and washing the hands and face before and after feeding the neonate, which will reduce the incidence of COVID-19 infection in neonates of COVID-19-positive mothers.
